# Development of a Cohort Analytics Tool for Monitoring Progression Patterns in Cardiovascular Diseases: Advanced Stochastic Modeling Approach

**DOI:** 10.2196/59392

**Published:** 2024-09-24

**Authors:** Arindam Brahma, Samir Chatterjee, Kala Seal, Ben Fitzpatrick, Youyou Tao

**Affiliations:** 1 Department of Information Systems and Business Analytics College of Business Loyola Marymount University Los Angeles, CA United States; 2 School of Information Systems and Technology Claremont Graduate University Claremont, CA United States; 3 Department of Mathematics Seaver College of Science and Engineering Loyola Marymount University Los Angeles, CA United States

**Keywords:** healthcare analytics, eHealth, disease monitoring, cardiovascular disease, disease progression model, myocardial, stroke, decision support, continuous-time Markov chain model, stochastic model, stochastic, Markov, cardiology, cardiovascular, heart, monitoring, progression

## Abstract

**Background:**

The World Health Organization (WHO) reported that cardiovascular diseases (CVDs) are the leading cause of death worldwide. CVDs are chronic, with complex progression patterns involving episodes of comorbidities and multimorbidities. When dealing with chronic diseases, physicians often adopt a “watchful waiting” strategy, and actions are postponed until information is available. Population-level transition probabilities and progression patterns can be revealed by applying time-variant stochastic modeling methods to longitudinal patient data from cohort studies. Inputs from CVD practitioners indicate that tools to generate and visualize cohort transition patterns have many impactful clinical applications. The resultant computational model can be embedded in digital decision support tools for clinicians. However, to date, no study has attempted to accomplish this for CVDs.

**Objective:**

This study aims to apply advanced stochastic modeling methods to uncover the transition probabilities and progression patterns from longitudinal episodic data of patient cohorts with CVD and thereafter use the computational model to build a digital clinical cohort analytics artifact demonstrating the actionability of such models.

**Methods:**

Our data were sourced from 9 epidemiological cohort studies by the National Heart Lung and Blood Institute and comprised chronological records of 1274 patients associated with 4839 CVD episodes across 16 years. We then used the continuous-time Markov chain method to develop our model, which offers a robust approach to time-variant transitions between disease states in chronic diseases.

**Results:**

Our study presents time-variant transition probabilities of CVD state changes, revealing patterns of CVD progression against time. We found that the transition from myocardial infarction (MI) to stroke has the fastest transition rate (mean transition time 3, SD 0 days, because only 1 patient had a MI-to-stroke transition in the dataset), and the transition from MI to angina is the slowest (mean transition time 1457, SD 1449 days). Congestive heart failure is the most probable first episode (371/840, 44.2%), followed by stroke (216/840, 25.7%). The resultant artifact is actionable as it can act as an eHealth cohort analytics tool, helping physicians gain insights into treatment and intervention strategies. Through expert panel interviews and surveys, we found 9 application use cases of our model.

**Conclusions:**

Past research does not provide actionable cohort-level decision support tools based on a comprehensive, 10-state, continuous-time Markov chain model to unveil complex CVD progression patterns from real-world patient data and support clinical decision-making. This paper aims to address this crucial limitation. Our stochastic model–embedded artifact can help clinicians in efficient disease monitoring and intervention decisions, guided by objective data-driven insights from real patient data. Furthermore, the proposed model can unveil progression patterns of any chronic disease of interest by inputting only 3 data elements: a synthetic patient identifier, episode name, and episode time in days from a baseline date.

## Introduction

Chronic conditions are defined as conditions that last for 1 year or more, require continuous medical care, or limit the ability to perform daily activities. They pose significant health challenges and financial burdens in the United States and worldwide [1,2]. In the United States, 6 in 10 adults have at least 1 chronic condition, and 4 in 10 have 2 or more [1]. Worldwide, nearly one-third of adults worldwide have with multiple chronic conditions [2].

Among these chronic conditions, cardiovascular diseases (CVDs), defined by the World Health Organization (WHO) as a group of disorders of the heart and blood vessels, are a significant public health concern worldwide. The WHO reported that CVDs are the leading cause of death worldwide, with an estimated 17.9 million deaths from CVDs in 2019, accounting for 32% of all deaths worldwide [3]. The American Heart Association also reported that in 2020, there were 928,713 CVD-related deaths in the United States, making it the leading cause of death in the United States [4].

CVDs are characteristically chronic, with a pattern of progression through many stages and episodic instances over time. Moreover, they are often associated with various comorbidities such as congestive heart failure (CHF), myocardial infarctions (MI), coronary heart disease, angina, stroke, and other complications [4]. For managing complex chronic conditions such as CVDs with long progression cycles, a detailed understanding of the progression pattern of disease states over time is essential [5,6]. This knowledge not only facilitates clinical decision-making but also enables hospitals to allocate their resources better [5]. Such models developed using representative population data can enable physicians to compare a patient’s progress with the patterns in the base population model to evaluate whether an intervention strategy reduces the transition probabilities between states of interest [7].

When dealing with chronic diseases, physicians often adopt a “watchful waiting” strategy and actions are postponed until information from an evolving clinical scenario is available [8]. However, data-driven clinical decision support tools with the ability to generate transition probabilities and progression paths can allow the development of effective intervention strategies at a cohort level, leading to better treatment outcomes. For this purpose, researchers have proposed and developed various quantitative disease progression models based on mathematical functions to understand the progression patterns of complex chronic diseases [6]. Quantitative disease progression models can be applied to track and describe the changes in disease progression over time and enable physicians to continually monitor and tailor treatment strategies and interventions [5,6]. Such models have significantly contributed to managing chronic progressive diseases such as Parkinson disease [5] and can be a critical precursor to policy development in cancer control [9]. Work on quantitative disease progression modeling has been found addressing various conditions such as Alzheimer disease and glaucoma [10,11], chronic kidney disease [12], abdominal aortic aneurysm [13], multiple sclerosis [14], and cardiovascular disorders, such as hypertrophic cardiomyopathy [15].

Among these quantitative disease progression models, stochastic models such as Markov models are widely applied to analyze disease processes [16-18]. Soper et al [19], for example, studied the dynamic progression of COVID-19 during the course of hospitalization using a continuous-time hidden Markov model. A Markov process can be constructed as a discrete-time Markov Chain (DTMC) model when the observations of the events are captured at a fixed recurring interval of time. However, DTMC’s approach to time quantification may not be suitable for diseases requiring frequent monitoring over short periods and observation over extended spans ranging from years to decades [20]. In contrast, events can be modeled as a continuous-time Markov chain (CTMC) when the recurring periods of observations are not fixed. CTMC models offer a realistic approach, supporting state transitions at any instant in a continuous time scale [21,22]. This motivated our study to adopt CTMC as the stochastic modeling method to unveil time-variant progression patterns of CVDs. There are other stochastic modeling approaches in the disease domain. We have provided a comparative analysis of the CTMC approach with 5 other approaches in Multimedia Appendix 1.

To better understand the extent of work in CTMC applications in disease progression modeling, we conducted a literature search (period: 2000-2023) and analyzed the studies that applied CTMC—an advanced stochastic modeling method—to model disease progression. The details of the paper search process and the results are described in Multimedia Appendix 2. We found only 7 papers that used the CTMC approach to model progressions of various diseases. The diseases studied in these papers are chronic or complex, requiring long-term observation and management, such as fibrosis, myelodysplastic syndromes, diabetic foot complications, Alzheimer disease, and chronic kidney disease. Each of these papers addresses a gap in understanding disease progression. For example, Meyer et al [23] aim to estimate progression time in fibrosis stages; Nicora et al [24] used simulation to generate longitudinal event data from cross-sectional patient data and build a CTMC model to arrive at the transition probabilities representing various stages of myelodysplastic syndrome progression. Begun et al [25] note the lack of knowledge about diabetic foot progression dynamics.

This analysis indicates that although researchers clearly recognize the need and clinical benefits of time-variant stochastic models to understand the progression of chronic diseases, only a few papers have applied the CTMC approach to model disease progression, and notably, none applied CTMC to CVDs. Most of these papers are limited in scope, focused on the disease progression encompassing a limited number of states of a single disease, and do not provide any framework for application to other chronic diseases. Furthermore, within the scope of CVDs, to the best of our knowledge, there is no paper providing actionable cohort-level analytical tools based on a comprehensive, 10-state, CTMC model to unveil complex progression patterns from real-world patient data and support clinical decision-making. Thus, our paper aims to address this crucial limitation in extant health informatics literature. Specifically, our proposed CTMC model aims not only to offer tools for clinicians to make informed intervention and treatment decisions for patients with CVDs, based on objective data-driven insights from real patient data, but also be adaptable for studying the progression of other chronic and complex diseases that require monitoring over time.

Novelties and contributions of this paper include the following:

- Application of advanced stochastic modeling, CTMC, to real-life patient cohort data can uncover new knowledge about CVD progression patterns and transition probabilities.
- Physicians can potentially use the digital data visualization system as an eHealth cohort analytics tool in CVD management, and we have found 9 impactful application use cases externally validated by a panel of 7 cardiologists.
- The proposed model can unveil progression patterns of any chronic disease of interest by inputting only 3 data elements: a synthetic patient identifier, episode name, and episode time in days from a baseline date. This would allow future researchers to generate and study disease progression patterns for other chronic diseases.
- The CVD transition probabilities can help health care administrators calculate the anticipated patient mix at different CVD states for a future planning period. This can facilitate predictive resource planning, improved patient care, and cost savings.
- Results are reproducible and extendable as the data, code, and development framework are shared with the audience via a web-based repository.

## Methods

### Data

The data applied in this research were from a multicenter cohort study implemented by the National Heart Lung and Blood Institute (NHLBI), collected from 9 epidemiological studies (Sleep Heart Health Study) on heart and respiratory diseases comprising 5804 patient records and 4839 CVD episodes associated with 1274 patients [26]. These longitudinal data were collected in 3 cycles across 16 years, with the first collection in 1995 and 2 subsequent collections between 1995 and 2003. CVD events, including death, were tracked until 2011. The inclusion criteria of the cohort members were aged 40 years or older, with no history of sleep apnea treatment, no tracheostomy, and no current oxygen therapy.

Table 1 provides a snapshot of occurrences of various CVD episodes or states for 2 patients (patients IDs 200453 and 201195) randomly selected from the NHLBI data used in this research. CVD “states” in our model represent specific CVD events or episodes in the patient’s history. For this reason, in our model, we define CVD states as “episodic” states or events.

It is noteworthy in the above table that multiple CVD events occurred on the same day. These are examples of comorbidity and multimorbidity in real life for patients with CVD. In the Markov process, 1 patient can be only in 1 state at a time. Hence, for the same patient, every instance of comorbidity and multimorbidity event occurring simultaneously is defined as a single Markov state. Many patients can be in any of these Markov states for a patient population with CVD, but 1 patient can only be in 1 state at any given time. Based on this Markov principle and the time of occurrence of patients’ CVD episodes in our dataset, we began with 14 unique CVD Markov states as found in our data. However, 4 states were dropped as their occurrences were negligible compared to the others. This led us to a final set of 10 unique CVD states for further development of our model. The details of the record counts in the dataset for all 14 states are presented in Multimedia Appendix 3.

**Table 1:** Snapshot of cardiovascular disease episodes of 2 patients from the National Heart Lung and Blood Institute dataset.

Patient ID and episode name		Episode time (days from baseline)
**200453**		
	Myocardial infarction	572
	Congestive heart failure	2064
	Congestive heart failure	2562
	Myocardial infarction	2562
	Congestive heart failure	2593
**201195**		
	Congestive heart failure	1343
	Congestive heart failure	1426
	Angina	3086
	Congestive heart failure	3086
	Myocardial infarction	3086
	Congestive heart failure	3143
	Congestive heart failure	3183

### Modeling Assumptions and Approximations

The modeling assumptions and approximations can be generalized into 2 categories—Markovian assumptions and CVD data–related assumptions. They are addressed in detail below.

1. Finite number of Markovian states: There is a limited or finite number of possible states. States are collectively exhaustive and mutually exclusive. In CVDs, the same patient cannot be in 2 Markov states simultaneously. In our dataset of actual patients with CVD, we have found multimorbidity situations where a patient can have multiple events simultaneously. To comply with this Markovian rule, we have deliberately treated such multimorbidity events as single and unique Markov states (eg, simultaneous occurrences of angina and MI is defined as a separate unique state designated as “ANMI”).
2. Memory-less property of Markovian states: Markov states do not retain the memory of previous cycles or information from previous states leading to the future state. For example, in our model, we assume that all patients in the MI state, at the same time, have the same probability of transitioning to the angina state, irrespective of their previous history or path of reaching the previous MI state.
3. No immediate transition to the same state: Our dataset has patient instances where a patient reported the same CVD episode (eg, stroke) occurring consecutively with a time gap. In such cases, we preprocessed the data to combine them into 1 continuous state, as due to the nature of CVDs, it might not be accurate to assume that the patient had a complete remission during the interim period. For example, if a patient reported a stroke episode in day number 100 and reported a consecutive stroke again in day number 110, we assume that the patient was in the stroke state for the interim period of 10 days as well. In other words, the patient was continuously in the stroke state from 100th day to 110th day and this is counted as a single continuous disease state with longer wait time during the data preprocessing. However, if after a stroke the patient had an angina episode had a stroke again, our model state diagram would show a return to a stroke state again after the angina episode.
4. Exponential distribution approximation of wait times: Several studies and reviews support the use of exponential distribution approximations in time-to-event modeling for CVDs. For example, Sullivan [27] highlights that the exponential distribution is a common choice for modeling the time to cardiovascular events. In the application of the CTMC process in disease progression modeling for CVDs, holding time or wait time in a given state is approximated to be exponentially distributed.

### Stochastic Modeling Theoretical Underpinnings

A stochastic process is a collection of random variables indexed by a variable *t*, usually representing time. A Markov Chain or Markov process is a stochastic model representing time-dependent cyclic processes. The primary advantage of a Markov process is the ability to describe, in a mathematically convenient form, the time-dependent transitions between health states [28].

**Figure 1:**
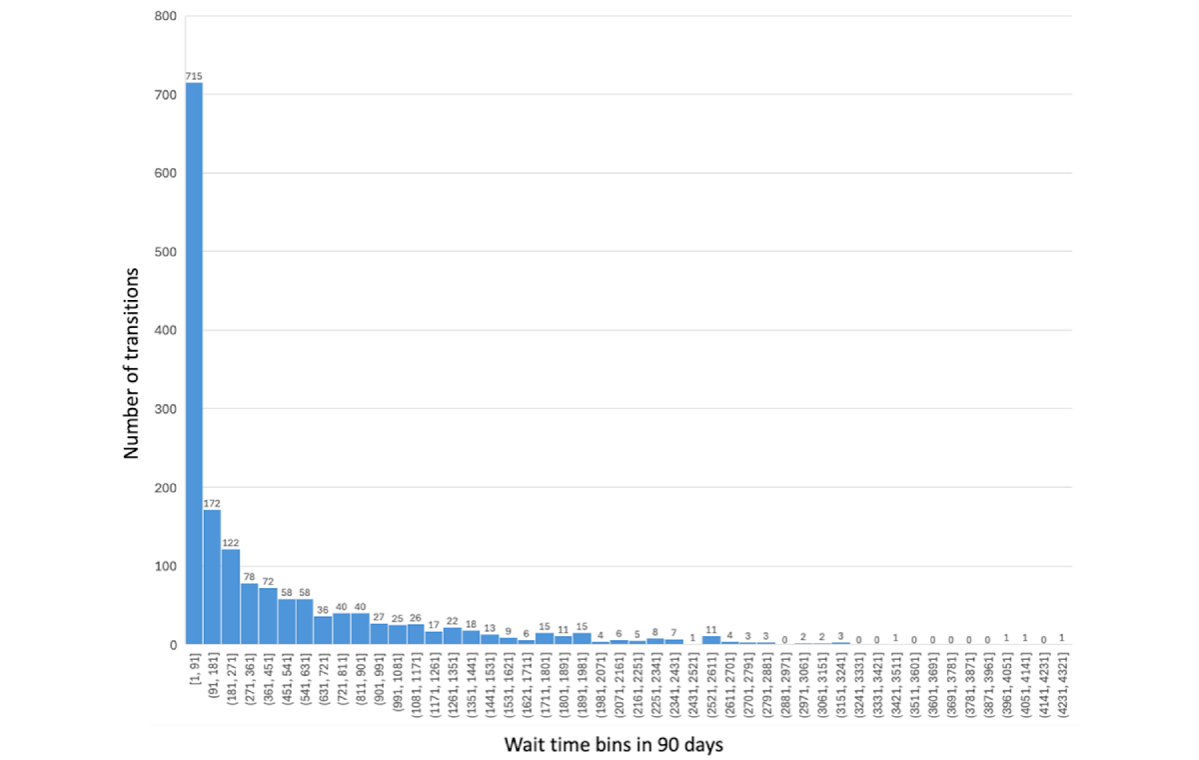
Wait time–frequency distribution in the research data set.

As discussed in the previous section, in a disease progression process, the random time variable *t*—“wait time” or “hold time”—is known to follow an exponential distribution. As a validation, we computed our dataset’s “wait time” frequency distribution and found it to have an approximate exponential frequency distribution pattern (Figure 1), although literature indicates that it is not strictly followed in the medical field, but necessary for modeling disease progressions successfully [29].

### Operational Behavior of CTMC

The operational behavior of a process modeled after CTMC can be described in the following steps, where a patient:

1. Stays *X_i_* units of time in state *i*, where *X_i_* is the random variable with an exponential distribution. The unit of time was measured in days in our study.
2. Jumps to a random next state *j* in a single step with probability *P(i,j)*
3. The behavior repeats across subsequent jumps.

Mathematically, CTMC can be defined by a tuple of 2 matrices: [S, R], where S is a matrix of *s* number of countable CTMC states and R is a transition rate matrix of (*s×s*) size. The value of R(*i,j*) equals the rate at which a patient moves from state *i* to state *j* in 1 step. This R matrix is also known as the generator matrix (denoted by *Q* in later sections), which we will explain in the following subsection.

### Generator Matrix—Q

The generator matrix, a fundamental matrix in CTMC calculations, is known by many names, such as infinitesimal generator matrix, transition rate matrix, intensity matrix, *Q*-matrix, etc. The generator matrix represents the rate (eg, number of transitions per day) at which transitions happen between states in 1 step. We denote it as *Q*-matrix with its elements as *q_i,j_*, where



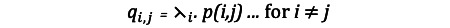





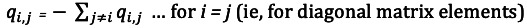



For diagonal matrix elements, *q*(*i,i*) is the negative sum of the rest of the row so that the row sum of each row is equal to zero. There are several methods of calculating the generator matrix. For this study, we applied the Maximum Likelihood Estimator method, which Metzner et al [30] discussed in their paper named “Generator Estimation of Markov Jump Processes.” In this method, for each state *i* of a finite number of states s, the generator matrix is computed as follows:

1. Calculate: *n_i.j_* = total number of transitions between state *i* to *j* for *i≠j*
2. Calculate: *r_i.j_* = total wait time or hold time at state *i* before transitioning to *j* for *i≠j*
3. Calculate: 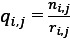

4. Place the negative of the row sum of nondiagonal positions as the diagonal entry so that the row sum of each row is zero. An infinitesimal generator matrix generates a continuous-time Markov process if and only if all off-diagonal entries are nonnegative and the sum over each row equals zero [30].

For example, if the current disease state is MI, as per our data, there are 31 cases (*n_i,j_*) where patients have transitioned from MI to CHF state in 1 step. During the hold time or wait time computations, we found that these patients, involving 31 MI-CHF transitions, waited a total of 16,633 days (*r_i,j_*) in the MI state before jumping to the CHF state. From these data points, *q_i,j_* (*i*=MI and *j*=CHF) can be calculated as 31 ÷ 16,633, which is 0.001864. This *Q*-matrix value is used to calculate the CTMC transition probability from MI to CHF at time *t* in the next step of the process.

### CTMC Transition Probabilities

Transition probabilities from state *i* to *j* after waiting time *t* at state *i* are obtained by using the generator matrix, *Q*, computed earlier as *P_i,j_* (*t*)=*e^Qt^*, where the exponent of *e* is calculated by multiplying each element of *Q* matrix (or *q_i,j_*) by the value of time *t*. Continuing the computation example from the previous section, if a patient is at a current state MI for 90 days, the probability of the patient transitioning to state CHF at the current time (*t*=90 days) can be calculated using the above exponential function. The calculated transition probability value from the *P_i,j_* (90) matrix (*i*=MI and *j*=CHF) will be 4.53% (Table 2).

**Table 2:** Continuous-time Markov chain (CTMC) transition probability matrix from state *i* to state *j* at a time of 90 days.

Beginning state	Immediate next state (%)										
	MI^a^	Stroke	Death	CHF^b^	CHMI^c^	CHST^d^	Angina	CHANMI^e^	ANMI^f^	CHAN^g^	Total
MI	0.63	40.18	19.60	4.53	1.15	5.47	20.23	1.74	1.27	5.21	100
Stroke	0.64	40.60	18.18	4.51	1.17	5.63	20.85	1.78	1.29	5.36	100
Death	0	0	100	0	0	0	0	0	0	0	100
CHF	0.67	22.75	13.84	33.27	1.75	3.14	14.51	1.96	1.55	6.55	100
CHMI	0.69	33.54	18.33	5.31	1.16	7.93	25.19	1.72	1.25	4.87	100
CHST	0.53	23.90	43.58	7.05	0.67	11.35	8.72	0.93	0.74	2.52	100
Angina	0.76	23.91	19.58	5.64	1.12	11.43	30.49	1.62	1.17	4.29	100
CHANMI	0.69	32.67	17.17	6.50	1.54	6.28	23.52	2.37	1.33	7.94	100
ANMI	0.64	39.05	18.56	4.85	1.29	5.14	19.94	1.88	1.34	7.32	100
CHAN	0.72	26.71	16.45	6.11	1.99	4.57	19.69	2.37	1.51	19.88	100

^a^MI: myocardial infarction.

^b^CHF: congestive heart failure.

^c^CHMI: congestive heart failure and myocardial infarction.

^d^CHST: congestive heart failure and stroke.

^e^CHANMI: congestive heart failure, angina, and myocardial infarction.

^f^ANMI: angina and myocardial infarction.

^g^CHAN: congestive heart failure and angina.

### Model Development Technical Process

**Figure 2:**
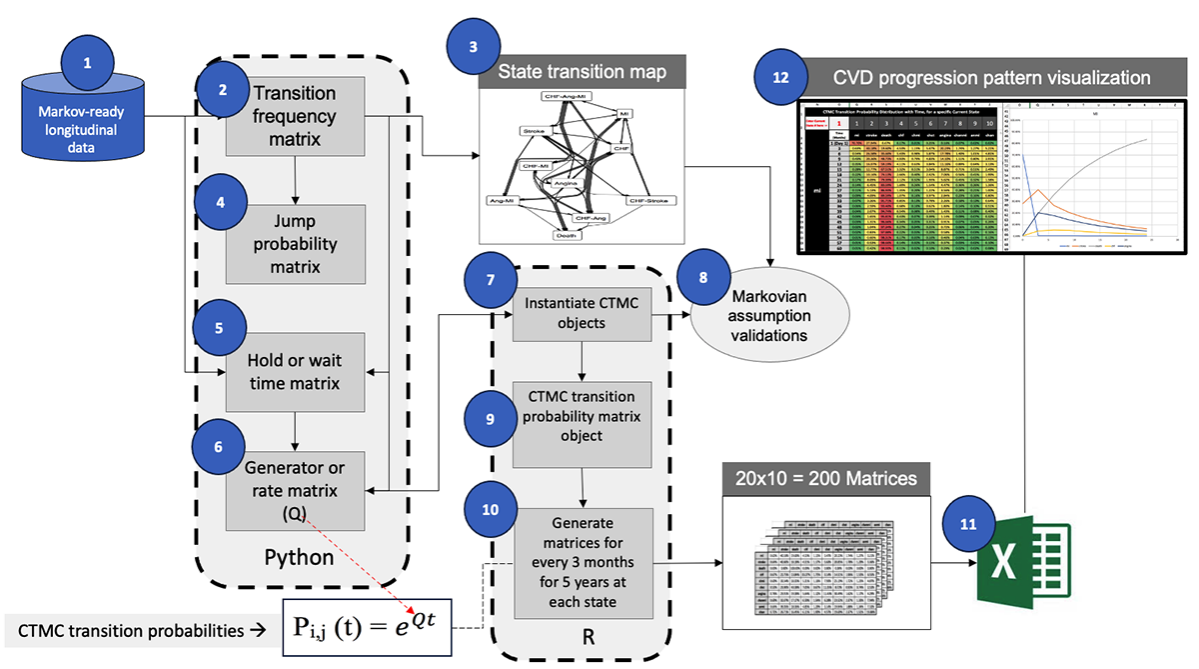
Development process for disease progression pattern generation and digital visualization. the circled numbers indicate the sequence of development steps and outputs. CTMC: continuous-time Markov chain; CVD: cardiovascular disease.

At this point, we want to highlight that, unlike the machine learning approach where the event probabilities are derived by training machine learning algorithms with historical data (supervised training), our approach uses a Markov Model, CTMC, which is a mathematical stochastic method to determine event probabilities and does not involve any sorts of training mechanism. Figure 2 illustrates our method used to generate a CTMC-based disease progression model for the CVDs. We have used all publicly available, open-source software tools and libraries for server-side module development (Python, Python Software Foundation, and R, R Foundation) and the widely used Microsoft Excel for front-end visualization artifacts. The *Results* section discusses further details.

### Deployment and User Interaction

**Figure 3:**
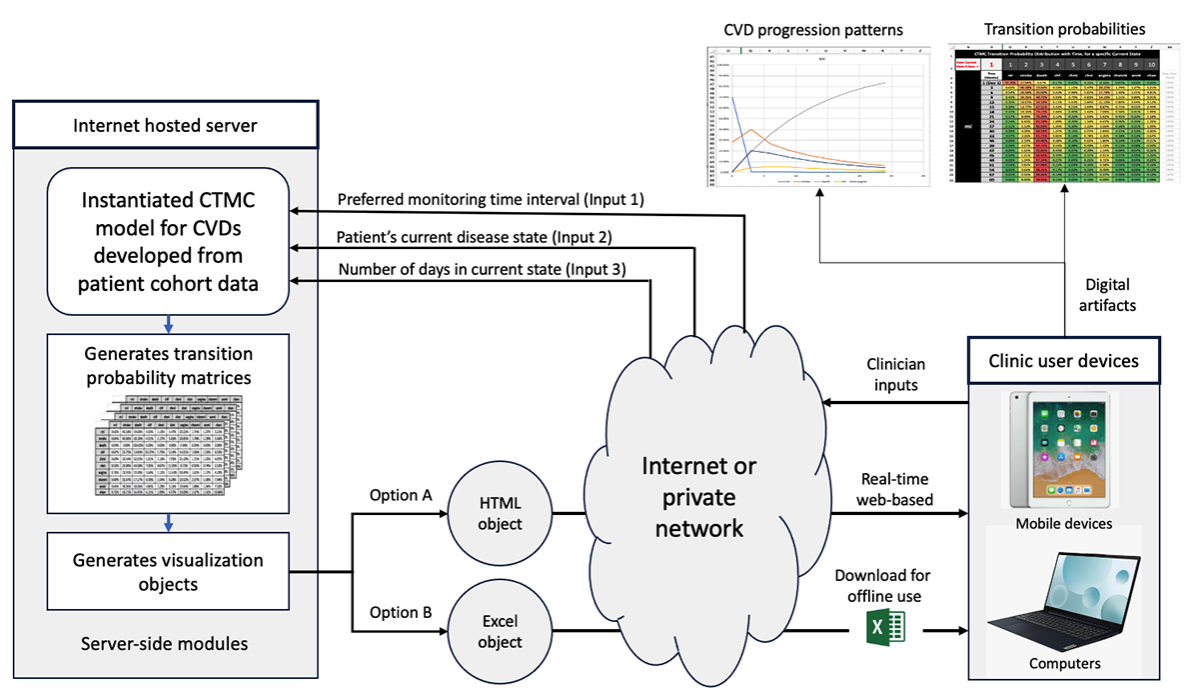
Proposed architecture for deployment of our system as an internet-based eHealth application for CVD clinicians with configurable monitoring time intervals. The architecture allows for synchronous web-based use (option A) as well as offline use via prior Excel (Microsoft Corp) artifact download (option B). CTMC: continuous-time Markov chain; CVD: cardiovascular disease.

Our developed system can be deployed as an eHealth solution for CVD clinics. In Figure 3, we present the high-level architecture of the proposed deployment configuration. Our proposed deployment design provides end users with the flexibility to use the system in real-time with an active internet or network connection using browsers, as well as the option of downloading the outputs as Excel artifacts and using them offline. We have provided the data, code, and Excel artifact (option B) via the web-based Mendeley repository [31] for reproducibility and reusability.

### Ethical Considerations

As discussed in this paper, cohort-level disease progression analytic tools are useful in many strategic clinical decisions. However, these are data-driven methods. Hence, ethical considerations applicable for data-driven clinical decision-making systems are important. Such considerations might include, but are not limited to, ensuring accuracy, bias-removal, fairness and equity, patient autonomy and consent, transparency and accountability, and privacy and data protection.

A Data Access and Use Agreement (the “DAUA”) is executed between The Brigham and Women’s Hospital, Inc., through its Division of Sleep and Circadian Disorders (“BWH”) and Arin Brahma (“Data User”) to facilitate access to and use of the de-identified sleep study and related covariate data originating from past NHLBI-funded research studies (the “Data”), by third-party researchers to conduct sleep research in accordance with NHLBI and BWH policies, procedures, and to the extent permitted by its Institutional Review Board (IRB) and institutional policies. The Data agreement can be made available by the authors upon request when deemed appropriate. The author has also received approval for the use of the Data for Cardiovascular Disease (CVD) research from the Institutional Review Board of Claremont Graduate University, CA, USA (Protocol ID is 3351; 01/11/2019).

## Results

The results comprise the computational outputs from the method described in the previous section. They include the CVD Markov state model, various probability matrices, and the progression pattern visualization results. These are explained in detail below.

### CVD Progression State Model

**Figure 4:**
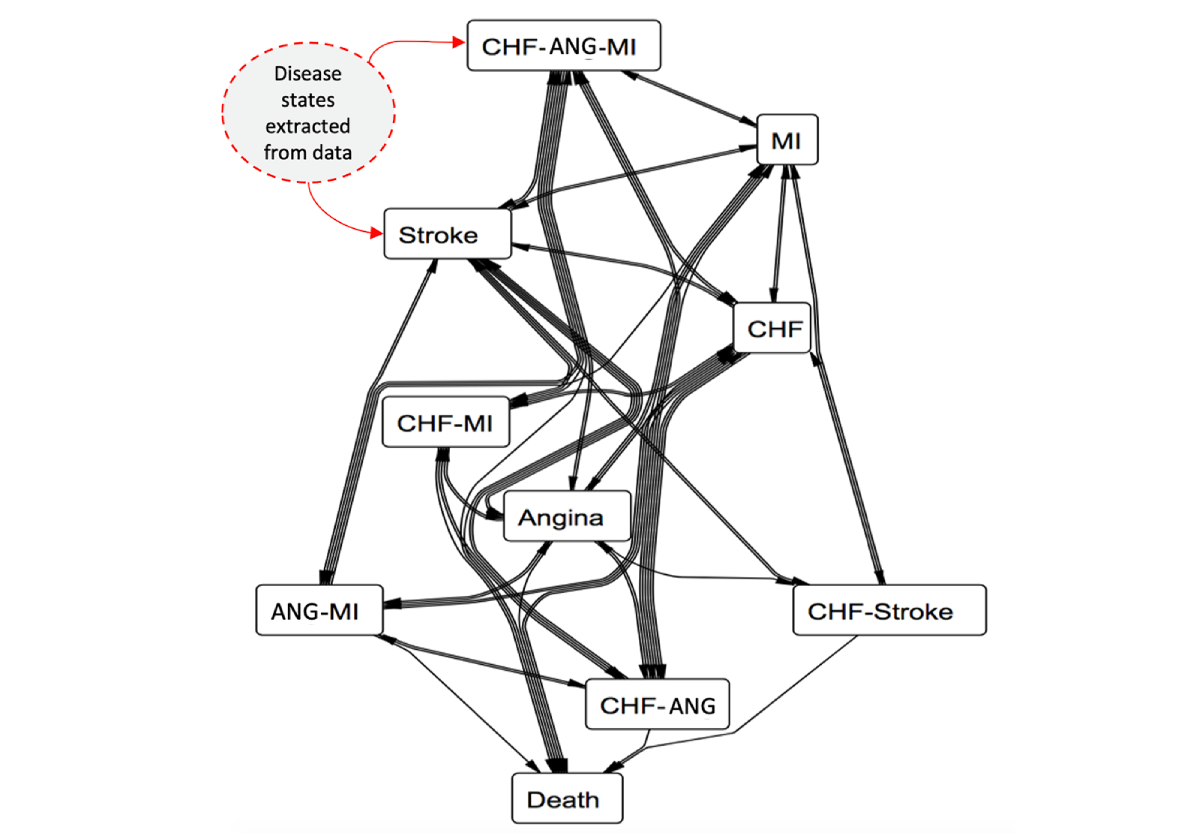
Markov state diagram (generated from jump frequency matrix). Ang: angina; CHF: congestive heart failure; MI: myocardial infarction.

We generated the Markov state transition graph (see Figure 4) based on the jump frequency matrix (Table 3) information. The direction of the arrows indicates the direction of disease transition, and the thickness indicates the proportion of the patients transitioning between the states.

Figure 4 visually illustrates the chronic nature of CVDs involving various morbidity, comorbidity, and multimorbidity states. It can be observed that the state death only has incoming arrows. This property makes death an “absorbing state” in a Markov model.

**Table 3:** Jump frequency matrix.

Beginning state	Immediate next state										
	MI^a^	Stroke	Death	CHF^b^	CHMI^c^	CHST^d^	Angina	CHANMI^e^	ANMI^f^	CHAN^g^	Total
MI	0	1	44	31	0	2	7	1	0	0	86
Stroke	10	0	74	18	2	9	3	6	7	2	131
Death	0	0	0	0	0	0	0	0	0	0	0
CHF	14	25	101	0	3	0	11	13	9	11	187
CHMI	0	1	8	6	0	0	2	0	0	1	18
CHST	1	6	3	4	0	0	0	0	0	0	14
Angina	9	11	12	39	1	1	0	7	7	14	101
CHANMI	7	4	10	19	1	0	13	0	4	4	62
ANMI	3	4	4	15	0	0	16	6	0	3	51
CHAN	1	2	6	19	2	0	8	5	1	0	44
Total	45	54	262	151	9	12	60	38	28	35	—^a^

^a^MI: myocardial infarction.

^b^CHF: congestive heart failure.

^c^CHMI: congestive heart failure and myocardial infarction.

^d^CHST: congestive heart failure and stroke.

^e^CHANMI: congestive heart failure, angina, and myocardial infarction.

^f^ANMI: angina and myocardial infarction.

^g^CHAN: congestive heart failure and angina.

^h^Not applicable.

### CVD State Jump Probabilities

The jump probability matrix is computed from the jump frequency matrix in Table 3 and displayed in Table 4. This matrix provides the probability of a patient jumping from state *i* to state *j*, without accounting for the effect of wait time or hold time at the current state on the transition probability. The cells with a transition probability of 0% indicate that no patient transitioned between those states.

**Table 4:** Jump probability matrix.

Beginning state	Immediate next state (%)										
	MI^a^	Stroke	Death	CHF^b^	CHMI^c^	CHST^d^	Angina	CHANMI^e^	ANMI^f^	CHAN^g^	Total
MI	0	1.2	51.2	36	0	2.3	8.1	1.2	0	0	100
Stroke	7.6	0	56.5	13.7	1.5	6.9	2.3	4.6	5.3	1.5	100
Death	0	0	100	0	0	0	0	0	0	0	100
CHF	7.5	13.4	54	0	1.6	0	5.9	7	4.8	5.9	100
CHMI	0	5.6	44.4	33.3	0	0	11.1	0	0	5.6	100
CHST	7.1	42.9	21.4	28.6	0	0	0	0	0	0	100
Angina	8.9	10.9	11.9	38.6	1	1	0	6.9	6.9	13.9	100
CHANMI	11.3	6.5	16.1	30.6	1.6	0	21	0	6.5	6.5	100
ANMI	5.9	7.8	7.8	29.4	0	0	31.4	11.8	0	5.9	100
CHAN	2.3	4.5	13.6	43.2	4.5	0	18.2	11.4	2.3	0	100

^a^MI: myocardial infarction.

^b^CHF: congestive heart failure.

^c^CHMI: congestive heart failure and myocardial infarction.

^d^CHST: congestive heart failure and stroke.

^e^CHANMI: congestive heart failure, angina, and myocardial infarction.

^f^ANMI: angina and myocardial infarction.

^g^CHAN: congestive heart failure and angina.

### CVD Continuous Time Transition Probabilities

Transition probabilities from state *i* to *j* after waiting time *t* at state *i* are obtained by using the generator matrix (*Q*) computed earlier as *P_i,j_ (t)=e^Qt^*, where the exponent of *e* is calculated by multiplying each element of *Q* matrix (or *q_i,j_*) by the value of time *t*. The generator matrix (*Q*) essentially represents the transition rates concerning time (eg, number of transitions per day) and is calculated before this step using the jump frequency and transition wait times.

If a patient is at a current state MI for 90 days, the probability of the patient transitioning to state CHF at the current time (*t*=90 days) can be calculated using the above exponential function. The calculated transition probability value from the *P_i,j_ (90)* matrix (*i*=MI and *j*=CHF) will be 4.53% (Table 2).

This is the final set of matrices of the CTMC model that leads to the digital data visualization of the temporal progression pattern of CVDs. In CTMC, the transition probability from state *i* to state *j* depends on the current value of time, as the transition probability changes depending on how long a patient is at the initial state *i*. For every such value of *t* (time), a different CTMC transition probability matrix exists for all state *i* to state *j* transitions.

### CVD Progression Pattern (Including Digital Data Visualization)

**Figure 5:**
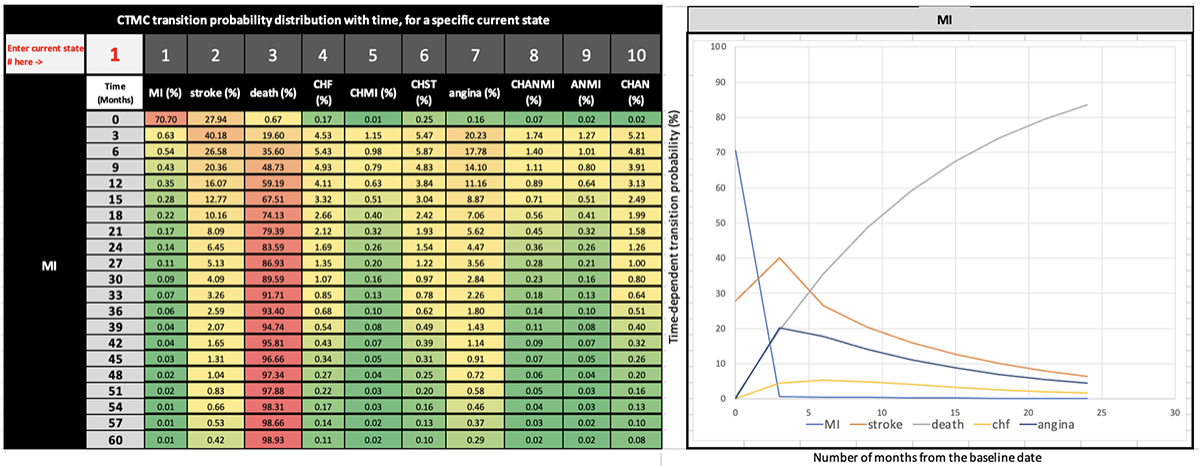
Digital data visualization: 3-month interval transition probabilities (left) and graphical progression pattern (right). Current state entered is 1 (MI)—best viewed in color. ANMI: angina and myocardial infarction; CHAN: congestive heart failure and angina; CHANMI: congestive heart failure, angina, and myocardial infarction; CHF: congestive heart failure; CHMI: congestive heart failure and myocardial infarction; CHST: congestive heart failure and stroke; CTMC: continuous-time Markov chain; MI: myocardial infarction.

**Figure 6:**
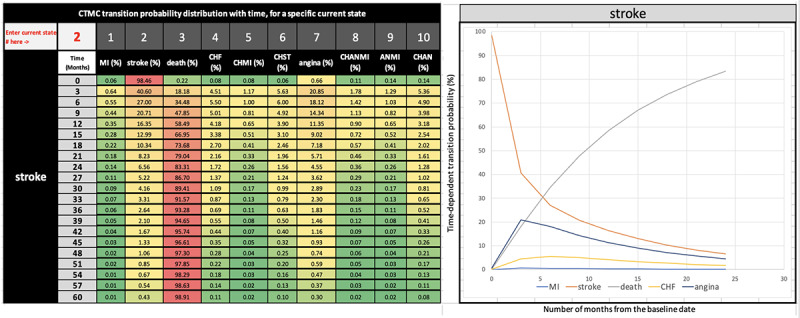
Digital data visualization: 3-month interval transition probabilities (left) and graphical progression pattern (right). Current state entered is 2 (stroke)—best viewed in color. ANMI: angina and myocardial infarction; CHAN: congestive heart failure and angina; CHANMI: congestive heart failure, angina, and myocardial infarction; CHF: congestive heart failure; CHMI: congestive heart failure and myocardial infarction; CHST: congestive heart failure and stroke; CTMC: continuous-time Markov chain; MI: myocardial infarction.

The CTMC transition probability matrix described above captures the transition probabilities at a specific recurring time interval scale of 3 months (90 days), as we assumed that, in general, CVD practitioners would review their patients’ progress every 3 months. However, the model allows the generation of the above matrix in any granularity (continuous) time scale, such as by days, weeks, or months. Our model autogenerated the CTMC probability matrices for every current state at a 3-month incremental progression scale for 5 years. This led to twenty 10-states by 10-states transition probability matrices. We developed a data aggregation and visualization method that automatically combines and organizes the probabilities from 20 matrices for each disease on a 5-year running time scale. This transformation presents each interstate transition probability matrix (Figures 5 and 6 left) in a “heat map” style with a value-based color scale (green is lowest, yellow is medium, and red is severe). Furthermore, this also automatically generates a time series trend graph (Figures 5 and 6 right) of transition probabilities that reveal the disease progression patterns graphically over 5 years when the current disease state of a patient is inputted by a clinician through the digital interface. Using this system, a CVD practitioner can visualize the temporal pattern and various “what if” scenarios to assist in decision-making regarding treatment or intervention strategies. For example, a CVD practitioner might be interested in knowing: if a patient had MI 3 months back (means current time *t*=3 months), what would be the probability of the patient transitioning to stroke or angina? From the following CTMC matrix generated for *t*=3 months, or 90 days by our model (Table 2), one can observe that if the current state of the patient is MI (rows), the probability of having a stroke is 40.18% (highest) and angina 20.23% (next highest). Hence, the CVD practitioner might recommend treatment or lifestyle interventions so that the risk of stroke or angina can be reduced.

### Analysis of CVD Progression Pattern

We explain our observations using an illustrative example (Figure 5—left and right), where the current CVD state of a hypothetical patient (representing population CVD progression pattern) is MI at *t*=1 (day 1 of month 1), the probability of staying in that state drops rapidly with time. In contrast, the probability of stroke, death, and CHF keeps increasing. Among these states, the probability of stroke starts with a higher probability of 28% at *t*=month 1. Continuing the analysis from Figure 5, the probability of death and angina starts at near zero but increases at a higher rate. At *t*=month 3, the probability of stroke, angina, death, and CHF peak at 40%, 20%, 20%, and 5%, respectively. At this point, if the patient transitions to stroke (40%), there is a 36% probability of death at *t*=month 6, compared to an 18% probability of angina and a 6% probability of CHF. If the patient escapes death and continues to be in the stroke state, then the probability of death crosses the 50% mark at *t*=month 9, and the death probability increases rapidly from then onwards. In 21 months, the death probability crosses the 80% mark, signifying a high probability of mortality.

Although the above findings meet the core objectives of this study, we performed further analysis of the dataset, which contributed additional interesting findings on CVD patterns. We use only 4 states—CHF, stroke, MI, and angina—for this analysis as they collectively account for 88.2% (231/262) of all CVD-related deaths (101/262, 38.6%; 74/262, 28.2%; 44/262, 16.8%; and 12/262, 4.6%, respectively). The details of this analysis are presented in Multimedia Appendix 4, and a summary of the key findings from this analysis is presented.

1. Transition rates and mean transition times: The fastest transition rate is from MI to stroke (mean transition time 3, SD 0 days, because only 1 patient had myocardial infarction to stroke transition in the dataset), and the slowest is from MI to angina (mean transition time 1457, SD 1449 days).
2. First CVD episodes: CHF is the most probable first episode (371/840, 44.2%), followed by stroke (216/840, 25.7%), angina (140/840, 16.7%), and MI (113/840, 13.5%).
3. Most dominant CVD episodes immediately after an episode: CHF is the most dominant episode after an occurrence of angina, whereas after CHF, stroke, and MI, death is the most dominant episode.

### Model Validation

As practiced commonly in validating empirical models, such as machine learning predictive models, one would ideally hold some testing data out to compare to a model built on training data. Fitzpatrick [31], in his paper “Issues in Reproducible Simulation Research,” states that the problem context of probabilistic models can often make this type of testing difficult. He discussed this aspect of stochastic model validations and laid a guideline for researchers working with such models. In the paper, Fitzpatrick refers to a multistage validation model from North and Macal [32] that includes validation of requirements, data, face, process, theory, and output. We used this model for internal evaluations during our model development cycles, as described in detail in Multimedia Appendix 5.

### Model Artifacts, Reproducibility, and Generalizability

One of the significant outputs of this research is the CTMC model embedded visualization artifact that CVD clinicians can use as a cohort-level decision-support tool for several applications and use cases as described in detail in Multimedia Appendix 6. The user interface of this 10-state model artifact has an input field (pointed out by the text “Enter Current State# here ” in Figures 5 and 6), where a clinician can be informed of the transition probability values and graphical progression patterns by entering the current CVD state of a cohort under investigation. This Excel artifact is self-contained, ready to use, and can be operated by having just the Microsoft Excel application on any computer. The artifact can be downloaded from the code repository [29].

Multimedia Appendix 7 provides more detailed information and instructions for future researchers interested in reusing these research methods, codes, and outputs to investigate the progression patterns of other chronic diseases or CVDs from different population datasets.

## Discussion

### Principal Findings

This research applies a well-established stochastic modeling—CTMC models on real-life patient cohort data to uncover previously unknown knowledge about CVD transition probabilities and complex progression patterns. The choice of CTMC overcame DTMC’s limitations on time quantification for disease progression modeling. There are other types of Markov models applied for stochastic modeling in the disease domain. The choice of appropriate modeling method is often driven by the model’s application goals (eg, patient-specific decisions vs cohort-level decisions). We have provided a detailed comparison of our choice of CTMC with 6 other methods in Multimedia Appendix 1. For complex chronic diseases such as CVDs, the availability of population-level transition probability matrices of all 10 disease states and respective progression patterns close a crucial gap in the literature and opens the door for future research on the disease progression of CVDs.

We find that the transition from MI to stroke has the fastest transition rate (mean transition time 3, SD 0 days, because only 1 patient had myocardial infarction to stroke transition in the dataset), and MI to angina is the slowest (mean transition time 1457, SD 1449 days). CHF is the most probable first episode (371/840, 44.2%), followed by stroke (216/840, 25.7%). This result reflects the epidemiological characteristics of our study cohort. However, if a dataset from a different cohort is inputted in our model, the patterns might differ.

### External Validations of Clinical Applications and Usefulness

The research also has many practical implications. We organized an expert panel of 7 practicing cardiologists and conducted a combination of presentations, open-ended interviews, and structured web-based surveys to validate the applications and usefulness of our model. We have discussed the methods, results, and analysis of this external validation process in detail in Multimedia Appendix 6. Here, we are presenting 9 clinical applications and use cases that were evident from the interviews and survey.

1. This tool can help cardiologic clinics understand the effectiveness of their intervention strategies related to quality metrics, such as blood pressure thresholds for interventions.
2. Comparing the clinic-level transition rates with population-level rates built into this tool, clinics can identify a specific cohort that has significantly higher rates for certain transitions (eg, angina-to-death) and then back-identify the patients for analysis of variables leading to such outcomes.
3. Clinics can use the transition probabilities generated by this tool based on their patient cohort data to compute the patient state mix for a future period. This can allow predictive resource planning, improved patient care, and cost savings.
4. This system can be populated with CVD episode data of various clinic cohorts and subcohorts and their patterns (frequency, transition rates, probabilities, etc) can be compared. This can reveal the trend differences concerning various cohort control features such as demographics, education, nutrition profile, treatment alternatives, etc.
5. Such intercohort benchmarking can delineate best practices concerning cost-effectiveness, manpower economy, and fund allocations (eg, Affordable Care Act incentives) when the candidate cohorts are selected from disparate clinics.
6. The utility can be further extended to understand epidemiological trends of different geographical and population segments, leading to valuable inputs for the health care policymakers and administrators enabling them to target specific regions based on pattern differences.
7. This tool can also help comparison of CVD characteristics at a national level, for example, some countries might have more stroke compared to others having more CHF. This can lead to necessary preventive measures and national health policies.
8. It can help identify temporal changes in CVD trends in patients because of shifts in major treatment paradigms (eg, prestatin vs statin period pattern changes)
9. This tool can be potentially integrated with clinic electronic health record systems to continually monitor temporal pattern shifts over time and send out automated notifications and reports based on preset quality metrics thresholds.

### Study of Impact of Various Risk and Control Factors on Epidemiological Trends

The clinical applications suggested by the expert panel point to the tool’s general ability to reveal epidemiological trend differences across various subcategories within a given cohort or across different cohorts. Such subcategories might include various population risk factor groups (age, gender, smoking behavior, education levels, etc), geographical attributes (urban, rural, coastal, inland, state, country, etc), treatment policies (quality metrics), interventions (medication or procedures), and so on.

**Figure 7:**
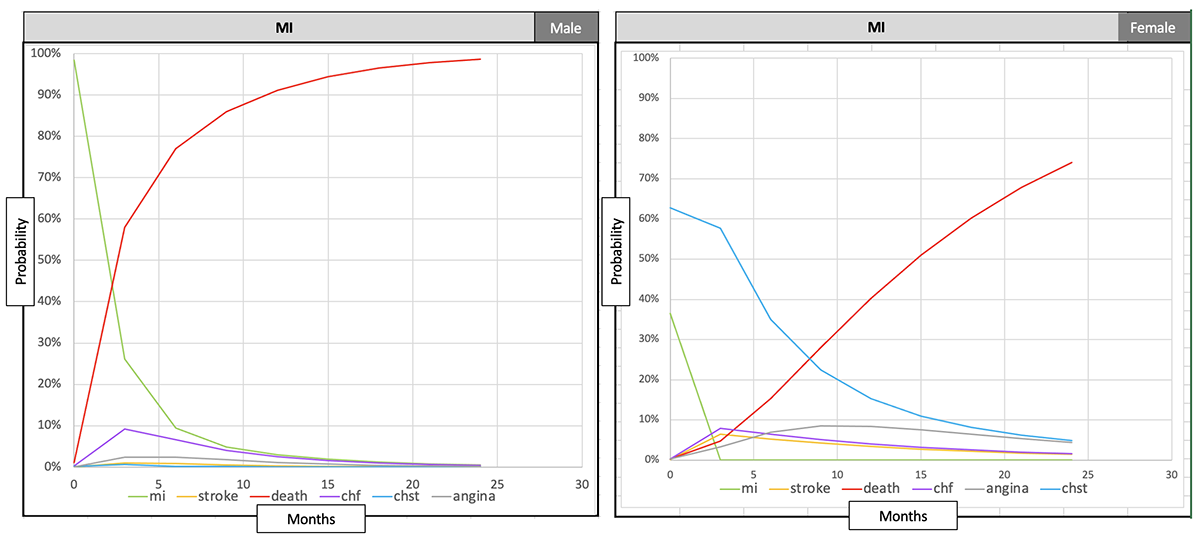
CVD transition probability pattern differences between male and female patients when the patients’ current state is “MI.” CHF: congestive heart failure; CHST: congestive heart failure and stroke; CTMC: continuous-time Markov chain; MI: myocardial infarction.

To demonstrate this key capability of our tool, we chose a population risk factor—“gender”—as an example and used the tool to visualize the CVD progression trend differences between male and female patients from our research cohort. To accomplish this, we partitioned the input dataset based on the “gender” subcategory. The data partitioning based on “gender” resulted in 278 male patients involved in 471 CVD episodes and 355 female patients involved in 592 CVD episodes. We then applied our research tool to generate 2 separate outputs corresponding to each of the genders. The visualization of the results (Figure 7) reveals distinct and interesting differences in the progression patterns between male and female patients within the cohort. In the following paragraph, we briefly outline some of the significant trend differences revealed between male and female patients when the current CVD state is “MI.”

First, we observe that the transition rate to state “death” (in red color) is significantly slower for the female patients compared to the male patients. The progression curve for females is noticeably flatter compared to their male counterparts. Thus, the female patients take almost 15 months to cross the probability threshold of 50%, whereas the male patients cross that threshold in just about 2.5 months. Another key observable difference is revealed in the progression pattern of the multimorbidity state “CHST” (congestive heart failure and stroke in blue color). The graph shows that female patients have more than a 50% probability of transitioning to “CHST” immediately following their current “MI” state over the next 4 months. In contrast, male patients have negligible probabilities of transitioning to “CHST” following the current “MI” state. Also, the probabilities of transitioning out of the current “MI” state (in green color) to any other state have distinctly different patterns between the male and female patient groups. Such trend comparisons between subgroups or different cohorts might trigger further study and analysis by the clinicians leading to improved treatment strategies, quality metrics, or interventions.

In the *Deployment and User Interaction* section, we have also provided a proposed deployment architecture that will allow health care providers to implement the solution as an eHealth application over the internet. Furthermore, our cohort-level clinical decision support system can be used for other chronic diseases to unveil their progression patterns, requiring only 3 data elements as inputs: a synthetic patient identifier, disease episode name, and episode time in days from a baseline date. This approach can encourage and facilitate further studies on disease progression patterns of other chronic diseases, not limited to CVDs by future researchers.

### Limitations

Our study has a few limitations. As per the Markovian assumptions, the process’s behavior after any cycle is solely based on its state within that cycle, indicating that it does not retain the memory of previous cycles. This assumption about the memory-less state is tenuous in medicine since a patient’s history may give the physician important information about their condition in the present and future. Hence, this is an approximation in our model. It is noted from the literature that although the Markovian assumption is necessary to model prognosis with a finite number of states, it is not followed strictly in medical problems [33]. Next, the data used for this research comprised CVD episode records of 1274 patients. The generalizability of this model will significantly improve with a larger number of diverse patients with CVD data. The CTMC approach has its limitations as well for patient-specific decision support applications. Since a patient is likely to be associated with a specific medical history, medication, interventions, demographic risk factors, etc, each of the Markov states must be unique for all such combinations. This makes the design and computation of the CTMC model very complex, less interpretable, and computationally heavy. Other Markov methods such as the Markov decision process can model patient-specific sequential treatment decision-making processes more efficiently. These limitations also lead to future opportunities for research. Since the framework and the tool developed in this research are disease agnostic, reproducibility-tested, and the reproducible package has been publicly shared, future researchers can use this tool to populate with episodic data of any chronic disease of interest and apply them to similar clinical use cases and applications. Additionally, to improve the generalizability of its application for CVDs, data from various other cohorts can be modeled and validated by simply reusing our model with new datasets.

### Conclusion

Stochastic disease progression models developed from fully observed real patient cohort data to compare a patient’s CVD progression with a population pattern can provide better intervention-based decision-making capabilities to physicians. However, such models do not currently exist. This research uses CTMC methods to develop a disease progression model from the population data of 1274 patients associated with 4839 CVD episodes across 16 years. This study unveiled distinct CVD progression patterns and characteristics from the fully observed longitudinal data of patient cohorts. The results are actionable with the code and data framework shared with the audience. The model also serves as an eHealth decision support tool with digital visualization of progression patterns and opens the door for many practical applications, including proactive resource planning at hospitals. Our study results are reproducible and extendable to other chronic diseases. Despite certain limitations, this research contributes significantly to the literature and possible practical clinical applications.

## Appendix

Multimedia Appendix 1 Markov model types applied in the disease domain.

Multimedia Appendix 2 Literature search process and results.

Multimedia Appendix 3 CVD states and their occurrences. CVD: cardiovascular disease.

Multimedia Appendix 4 Additional analysis of CVD patterns. CVD: cardiovascular diseases.

Multimedia Appendix 5 Model validation.

Multimedia Appendix 6 Cardiologist expert panel interviews and web-based survey.

Multimedia Appendix 7 Model reproducibility information.

## Abbreviations

ANMI: angina and myocardial infarction
CHF: congestive heart failure
CHST: congestive heart failure and stroke
CTMC: continuous-time Markov chain
CVD: cardiovascular disease
DTMC: discrete-time Markov chain
MI: myocardial infarction
NHLBI: National Heart Lung and Blood Institute
WHO: World Health Organization

## Data Availability

The master dataset was obtained from a publicly available source—Sleep Heart Health Study by the National Heart Lung and Blood Institute (NHLBI) [26]. The preprocessed version of the dataset used for the continuous-time Markov chain (CTMC) model development is available at the publicly accessible web-based Mendeley repository [29].
