# Supplementary material for: Development of a Cohort Analytics Tool for Monitoring Progression Patterns in Cardiovascular Diseases: Advanced Stochastic Modeling Approach
Source: JMIR Med Inform. 2024 Sep 24;12:e59392. doi: 10.2196/59392 (PMC11462104; doi:10.2196/59392)
Supplement: Multimedia Appendix 1 [file medinform_v12i1e59392_app1.docx]

**Table S1.** Various Markov model types in the disease domain.

| **Type of Markov Model** | **Model Goals** | **Data Conditions driving the Applicability of the models** | **Relevant Citations** |
| --- | --- | --- | --- |
| Markov Chain Monte Carlo (MCMC) | Estimating disease prevalence and incidence from incomplete data or cross-dataset validations | Incomplete or missing data, prior distributions, likelihood functions | Nijhuis et al [1] |
| Markov Decision Process (MDP) | Treatment of chronic disorders involving sequential decision process, in which a sequence of interrelated decisions or actions must be made over time | Requires the dataset to have action-state pair for every transition | Bennett and Hauser [2] |
| Partially Observable Markov Decision Process (POMDP) | POMDPs extend MDPs by maintaining internal belief states about patient status, treatment effect, etc. | Requires the dataset to have action-state pair for every transition and works well when data is noisy or there are missing observations | Bennett and Hauser [2] |
| Hidden Markov Model (HMM) | Predicting disease states from medical test sequences | Sequences of medical test results, where some states exist, but are not observable, such as intermediate states | Murphy [3] |
| Discrete-Time Markov Chain (DTMC) | Modeling the progression of chronic diseases like diabetes | State transition events are observed and captured at fixed time intervals | Beck and Pauker [4] |
| **Continuous-Time Markov Chain (CTMC) - Model used in this research** | **Modeling the progression of chronic diseases like CVDs** | **State transition events happen in unpredictable time intervals. They are observed and captured when the event occurs** | **Aalen and Gjessing [5]** |

**References in the Appendix:**

1. Nijhuis RL, Stijnen T, Peeters A, Witteman JC, Hofman A, Hunink MM. Apparent and internal validity of a Monte Carlo–Markov model for cardiovascular disease in a cohort follow-up study. Medical decision making. 2006;26(2):134-44.

2. Bennett CC, Hauser K. Artificial intelligence framework for simulating clinical decision-making: A Markov decision process approach. Artificial intelligence in medicine. 2013;57(1):9-19.

3. Murphy KP. Dynamic bayesian networks: representation, inference and learning: University of California, Berkeley; 2002.

4. Beck JR, Pauker SG. The Markov process in medical prognosis. Medical decision making. 1983;3(4):419-58.

5. Aalen OO, Gjessing HK. Understanding the Shape of the Hazard Rate: A Process Point of View. Statistical Science. 2001:1-14.
