# Supplementary material for: Development of a Cohort Analytics Tool for Monitoring Progression Patterns in Cardiovascular Diseases: Advanced Stochastic Modeling Approach
Source: JMIR Med Inform. 2024 Sep 24;12:e59392. doi: 10.2196/59392 (PMC11462104; doi:10.2196/59392)
Supplement: Multimedia Appendix 2 [file medinform_v12i1e59392_app2.docx]

To identify gaps in the literature on the use of Markov Chain models specific to disease progression modeling, we conducted a search using the search term ‘((“disease progression" OR "disease progression pattern" OR "disease progression analysis" OR “disease prediction" OR “disease " OR “disease progression modeling") AND (“Markov chain” OR "discrete-time Markov chain” OR “DTMC" OR "continuous-time Markov chain” OR “CTMC” OR “stochastic modeling” OR “stochastic model"))’ for the period 2000 – 2023. We further scanned the results manually for the correctness of relevance. This resulted in only 26 papers. Seven of these 26 papers involved CTMC (Table 1), and the rest discussed DTMC or other variants of Markov Chain methods for disease progression modeling.

**Table S1.** Summary of literature for studies of CTMC models in disease progression. CTMC: continuous-time Markov chain.

| **Pub**  **Year** | **Reference** | **# of Patients** | **Disease Modeled** | **Model or Framework Developed** |
| --- | --- | --- | --- | --- |
| 2023 | Meyer, Musante [1] | 218 | Fibrosis in patients with Nonalcoholic Fatty Liver Disease (NAFLD) and Nonalcoholic Steatohepatitis (NASH) | Developed a CTMC model to better understand and predict the progression of fibrosis in patients with NAFLD and NASH. |
| 2020 | Nicora, et al.[2] | 921 | Myelodysplastic syndromes (MDS) | Developed a CTMC model from patient cross-sectional data to calculate the transition probabilities that represent the patients’ evolution across various stages of MDS progression. |
| 2016 | Begun, Morbach [3] | 260 | Diabetic foot diseases | Developed a nine-state CTMC model was and quantified the mean times to transition in years and conditional probabilities for various transitions. |
| 2015 | Liu, Li [4] | Not specified | Alzheimer’s, Glaucoma, etc. | Developed algorithms to visualize and predict of disease progression in glaucoma and Alzheimer’s disease. |
| 2013 | Begun, Icks [5] | 2,097 | Chronic kidney disease | Developed a six-state CTMC model for chronic kidney disease progression. |
| 2011 | Leiva-Murillo, Rodrıguez [6] | 374, 955 | Mental disorders | Developed a Hidden Markov Model CTMC to visualize and predict the interaction and progression of diseases. |
| 2010 | Sweeting, Farewell [7] | 858 | Hepatitis C virus infection | Developed a multi-state Markov model for Hepatitis C virus disease progression. |
|  | This paper | 1274 | Cardiovascular Diseases | Developed a transition path model, a stochastic model based on CTMC, and a visualization artifact to help the clinicians compare a patient’s disease progression trend against the population pattern revealed by the model. |

References:

1. Meyer LF, Musante CJ, Allen R. A continuous-time Markov chain model of fibrosis progression in NAFLD and NASH. Frontiers in Medicine. 2023;10:1130890.

2. Nicora G, Moretti F, Sauta E, Della Porta M, Malcovati L, Cazzola M, et al. A continuous-time Markov model approach for modeling myelodysplastic syndromes progression from cross-sectional data. Journal of Biomedical Informatics. 2020;104:103398.

3. Begun A, Morbach S, Rümenapf G, Icks A. Study of disease progression and relevant risk factors in diabetic foot patients using a multistate continuous-time Markov chain model. PLoS One. 2016;11(1):e0147533.

4. Liu Y-Y, Li S, Li F, Song L, Rehg JM. Efficient Learning of Continuous-Time Hidden Markov Models for Disease Progression. Advances in neural information processing systems. 2015;28:3599-607.

5. Begun A, Icks A, Waldeyer R, Landwehr S, Koch M, Giani G. Identification of a multistate continuous-time nonhomogeneous Markov chain model for patients with decreased renal function. Medical Decision Making. 2013;33(2):298-306.

6. Leiva-Murillo JM, Rodrıguez AA, Baca-Garcıa E, Dıaz FJ, editors. Visualization and prediction of disease interactions with continuous-time hidden markov models. NIPS 2011 Workshop on Personalized Medicine; 2011. Granada, Spain.

7. Sweeting M, Farewell V, De Angelis D. Multi‐state Markov models for disease progression in the presence of informative examination times: An application to hepatitis C. Statistics in medicine. 2010;29(11):1161-74.
