# Supplementary material for: Development of a Cohort Analytics Tool for Monitoring Progression Patterns in Cardiovascular Diseases: Advanced Stochastic Modeling Approach
Source: JMIR Med Inform. 2024 Sep 24;12:e59392. doi: 10.2196/59392 (PMC11462104; doi:10.2196/59392)
Supplement: Multimedia Appendix 3 [file medinform_v12i1e59392_app3.docx]

**Table S1.** List of single, comorbidity, and multimorbidity CVD states. CVD: cardiovascular disease.

|  | **Episode Name** | **Abbreviation** | **Number of Occurrences** |
| --- | --- | --- | --- |
| 1 | Congestive Heart Failure | chf | 522 |
| 2 | Myocardial Infarction or Heart Attack | mi | 178 |
| 3 | Stroke | stroke | 270 |
| 4 | Angina | angina | 200 |
| 5 | Death from CVD | death | 262 |
| 6 | CHF and MI | chmi | 22 |
| 7 | CHF and Stroke | chst | 25 |
| 8 | Angina and MI | anmi | 99 |
| 9 | CHF and Angina | chan | 68 |
| 10 | CHF, Angina, and MI | chanmi | 88 |
| - | Angina and Stroke* | - | 4 |
| - | MI and Stroke* | - | 1 |
| - | Angina, MI, and Stroke* | - | 3 |
| - | CHF, Angina, MI, and Stroke* | - | 2 |
| * Dropped due to negligible occurrences in the data set | | | |
