# Supplementary material for: Development of a Cohort Analytics Tool for Monitoring Progression Patterns in Cardiovascular Diseases: Advanced Stochastic Modeling Approach
Source: JMIR Med Inform. 2024 Sep 24;12:e59392. doi: 10.2196/59392 (PMC11462104; doi:10.2196/59392)
Supplement: Multimedia Appendix 4 [file medinform_v12i1e59392_app4.docx]

This section discusses further observations on transition probabilities and mean transition times among the four dominant states—CHF, stroke, MI, and angina. We use only these four states in this analysis as they collectively account for 88% of all CVD-related deaths (CHF 39%, stroke 28%, MI 17%, and angina 5%). Table 1 presents an analysis of the state transition probability of CHF, strokes, MI, angina, and death.

**Table S1.** Patients at CVD states transitioning from immediate previous states. CVD: cardiovascular disease.

|  | % of STROKE Patients coming from various states | % of CHF Patients coming from various states | % of MI Patients coming from various states | % of Angina Patients coming from various states | % of Deaths coming from various states |
| --- | --- | --- | --- | --- | --- |
| mi | 3% | 35% | 0% | 33% | 19% |
| stroke | 0% | 20% | 30% | 14% | 32% |
| chf | 68% | 0% | 42% | 52% | 44% |
| angina | 30% | 44% | 27% | 0% | 5% |

A study of the above table leads to the following observations (Table 2). The analysis implies that CHF is the most frequently occurring previous state before MI, stroke, angina, and death, and angina itself is the most frequently occurring state before CHF.

**Table S2.** Current states vs. immediate previous states.

|  | A. Patients Current State | B. Most Frequently Occurring Immediate Previous State of A | % of Patients Directly Transitioned from B to A directly |
| --- | --- | --- | --- |
| 1 | Stroke | CHF | 68% |
| 2 | Angina | CHF | 52% |
| 3 | Death | CHF | 44% |
| 4 | CHF | Angina | 44% |
| 5 | MI | CHF | 42% |

As presented in Figure 1 below, further data analysis shows that the most frequently occurring first CVD episode is CHF (44%), followed by stroke (26%), angina (17%), and MI (13%). Figure 1 also illustrates the most frequently occurring CVD states immediately after the first episode of the patients.


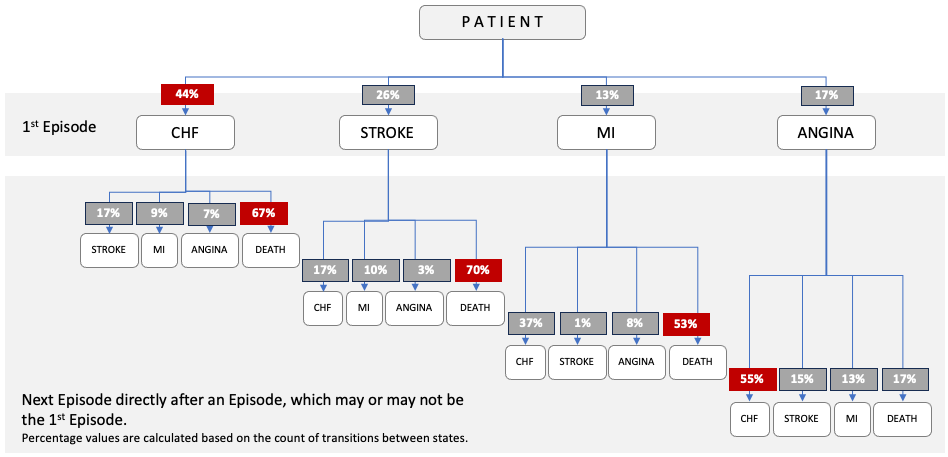


**Figure S1.** Probabilities of transition to the next CVD episode directly after an episode. CVD: cardiovascular disease.

Additionally, Table 3 presents the meantime of transitions in days between states. From this table, we find that the fastest time of transition is from MI (3 days) to stroke, and the slowest is from MI to angina (1457 days). This is also consistent with the observations of time transition rate or transition speed found from the Q-Matrix or transition rate table, wherein we observe that mi to stroke has the fastest transition rate and mi to angina is the slowest.

**Table S3.** Mean transition times between CVD states (in days). CVD: cardiovascular disease.

|  | mi | stroke | chf | angina | death |
| --- | --- | --- | --- | --- | --- |
| mi |  | 3 | 537 | 1457 | 132 |
| stroke | 1442 |  | 1337 | 151 | 465 |
| chf | 857 | 844 |  | 782 | 744 |
| angina | 487 | 890 | 1202 |  | 1134 |
