# Supplementary material for: Development of a Cohort Analytics Tool for Monitoring Progression Patterns in Cardiovascular Diseases: Advanced Stochastic Modeling Approach
Source: JMIR Med Inform. 2024 Sep 24;12:e59392. doi: 10.2196/59392 (PMC11462104; doi:10.2196/59392)
Supplement: Multimedia Appendix 5 [file medinform_v12i1e59392_app5.docx]

**Table S1.** North and Macal [1] framework used for CVD model validation. CVD: cardiovascular disease.

|  | **Validation Items** | **Validation Questions** | **Validations in this research** |
| --- | --- | --- | --- |
| 1 | Requirements Validation | Have the model requirements been properly specified for the problem at hand? | The model requires the process to be stochastic. Evidence from the literature shows that disease progression processes such as CVDs follow a stochastic progression model. |
| 2 | Data Validation | Have the data used to calibrate the model been properly collected and verified? | The data used for building this model is obtained from NHLBI (https://sleepdata.org/datasets/shhs). Numerous research publications using this dataset indicate the robustness of the data collection methods involved. |
| 3 | Face Validation | Do the model assumptions and outputs appear reasonable? | Model assumptions have been validated against Markovian assumptions. Reasonableness of the output has been validated through inputs from a practicing cardiologist. |
| 4 | Process Validation | Do the computational flow correspond to real-world processes? | The model is built using 4839 CVD episode data of 1274 real life patients. This itself is a validation of real-life correspondence of states and the computational flow. |
| 5 | Theory Validation | Does the model make valid use of the theory on which it is based | CTMC theories and principles has been used as the kernel theory in this research. The model has been constructed in a step-by-step manner with multi-step validations with the theory tenets and assumptions. |
| 6 | Output Validation | Do the model outputs compare to observed data? | An interview was conducted with a cardiologist, sharing the output of this model. Her expert opinion was solicited to relate the outputs with the observed data from her practice. The results are discussed below. |

For external evaluation or validation, we discussed the model and its findings with a practicing cardiologist from a very well-known hospital in the United States. We also conducted structured interview, and brief electronic survey with her to formally capture her inputs on our CTMC CVD progression model. The inputs and knowledge gathered from this effort are summarized below:

- While the disease progression probabilities and transition states are useful, the transition state “death” may not be used by the cardiologists. This is because if death is perceived to be a possible next transition state, it must be rigorously defended by clinical methods. Just a mathematical model may not stand up to the scrutiny demanded when any information about death as a possible transition state is linked to any possible clinical decision or action.
- The transition matrix probabilities as a guideline for non-fatal CVD events are certainly useful for cardiologists with respect to treatment strategies and decisions.
- Our NHLBI dataset period began from 1995 and updates continued until 2011. Year 2000 to 2002 was a major inflection point of CVD treatment paradigm due to introduction of statin class drugs. Hence it will be interesting to see if the transition model differs for the pre and post inflection point (future research scope).

Reference:

1. North MJ, Macal CM. Managing business complexity: discovering strategic solutions with agent-based modeling and simulation: Oxford University Press; 2007. ISBN: 0195172116.
