# Supplementary material for: Development of a Cohort Analytics Tool for Monitoring Progression Patterns in Cardiovascular Diseases: Advanced Stochastic Modeling Approach
Source: JMIR Med Inform. 2024 Sep 24;12:e59392. doi: 10.2196/59392 (PMC11462104; doi:10.2196/59392)
Supplement: Multimedia Appendix 6 [file medinform_v12i1e59392_app6.docx]

Objective: Establishing the external validity of applications and usefulness of the CVD Progression Model (CVD-PM) in cardiology clinical practice

Method: We organized an expert panel of seven practicing cardiologists and conducted a combination of presentations, open-ended interviews, and structured online surveys to meet the objectives. From the first interview we gathered a set of three potential application use cases of CVD-PM in cardiology clinical practice. We then presented those three use cases to other six cardiologists through the online survey platform for validation, along with an open-ended question on identifying additional applications from their practice experience. We present the details of the expert panel and the schedule of interviews in Table 1 below.

**Table S1.** Chronological list of interviews with expert panel details.

| **Cardiologist #** | **Date of Interview** | **Practice Specialty** | **Current Practice** | **Credentials** |
| --- | --- | --- | --- | --- |
| 1 | 5/14/24 | Heart Failure and Transplantation Cardiology | University of Miami Health Systems; Faculty - Miller School of Medicine, Miami, FL | MD, MPH; Harvard Medical School |
| 2 | 5/18/24 | Interventional Cardiologist | Choice Medical Group, Victorville, CA | MD, FACC, FSCAI; Universidad de Guadalajara Medical School |
| 3 | 5/19/24 | Consultant Physician and Interventionist, Cardiology | Millenium Clinic, Kolkata, India | MD, DNB, FICP, FACP, FRCP (London) |
| 4 | 5/22/24 | Cardiovascular Diseases | Desert Valley Medical Group, Victorville, CA | MD, FRCPC, FACP, FACC, FSCAI, RPVI |
| 5 | 5/26/24 | Interventional Cardiologist | AMRI Hospital, Kolkata, India; Asst. Prof, Cardiology, KPC Medical College, Kolkata, India | MD, MRCP(UK), PGD-Diabetes (BCU, UK), DM (Cardiology) |
| 6 | 5/29/24 | Cardio Thoracic & Vascular Surgery | Chief Cardiac Surgeon at Apollo Gleneagles Hospital, Kolkata, India | MD, MS, M.CH |
| 7 | 6/2/24 | Cardiovascular Diseases | Medical Director, Ascension Borgess Cardiovascular Lab, Clinical Professor of Medicine, WMU Homer Stryker MD School of Medicine, MI | MD, MPH, FACC, FSCAI |

The interviews and the CVD-PM clinical use survey:

Prior to the interview, an overview of the research was emailed to the panel members for prior reading. During the interview, we first conducted a further detailed walkthrough of the research using Zoom platform, followed by an online survey on the SurveyMonkey platform. For each of the first three online survey questions (Q1 – Q3, see Figures 1 - 3), we presented a realistic example of a clinical situation, followed by a multiple-choice question. The fourth question (Q4, see Figure 4) is open-ended and allows the cardiologist to identify additional application use cases from their practice experience. On an average the sessions were 45 minutes long and were recorded in the Zoom cloud platform, with permission from the panel members. In the following section, we present screenshots from the online survey.

**Figure S1.** Question #1.


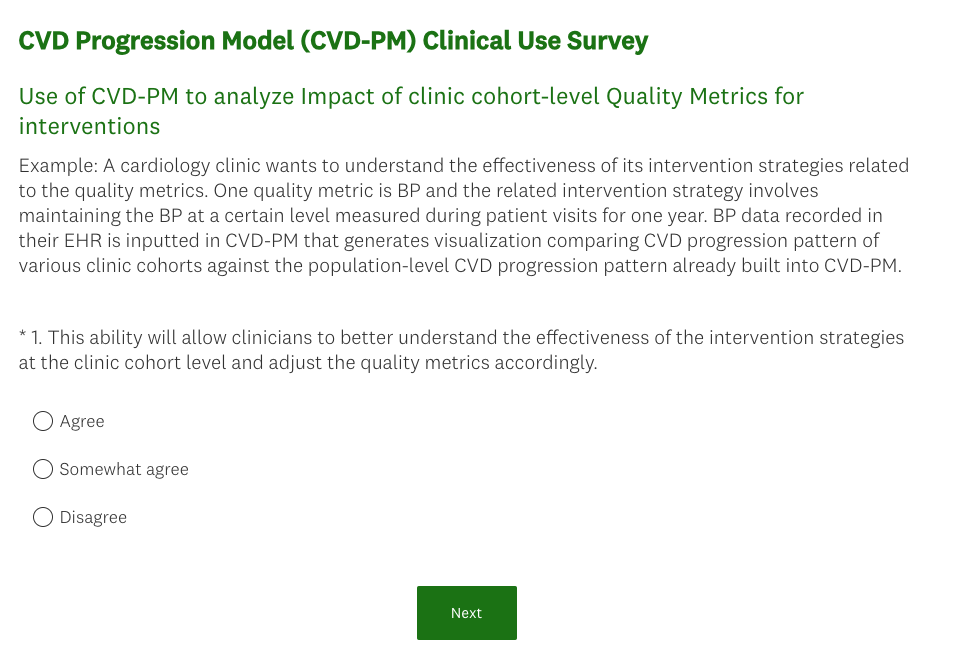


**Figure S2.** Question #2.


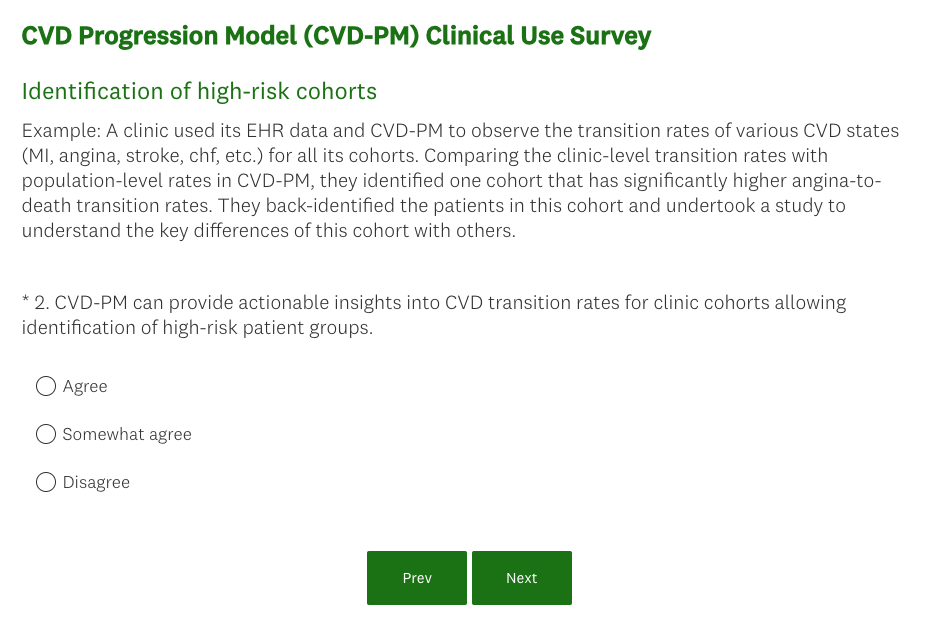


**Figure S3.** Question #3.


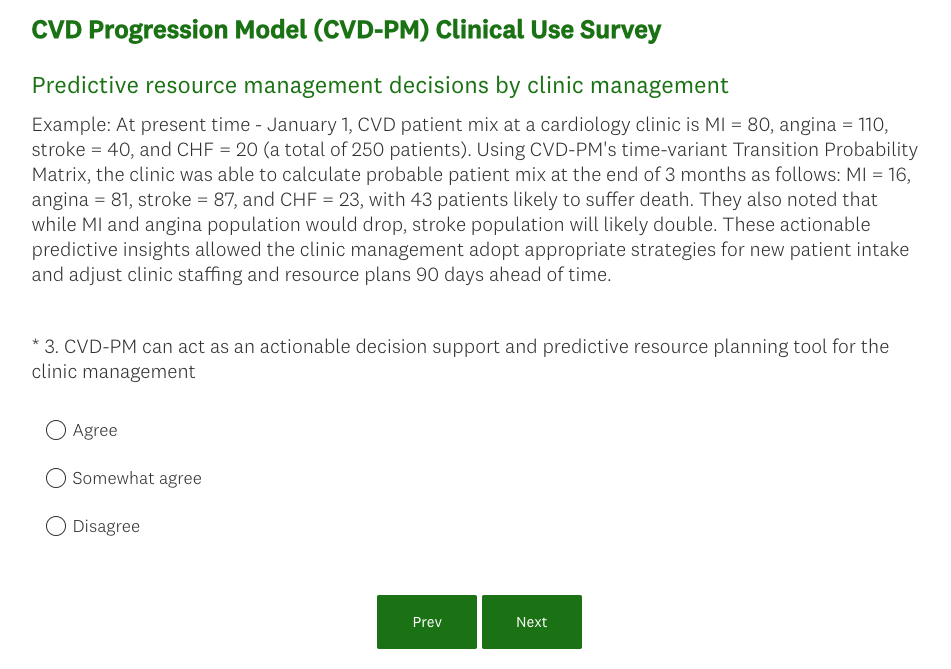


**Figure S4.** Question #4.


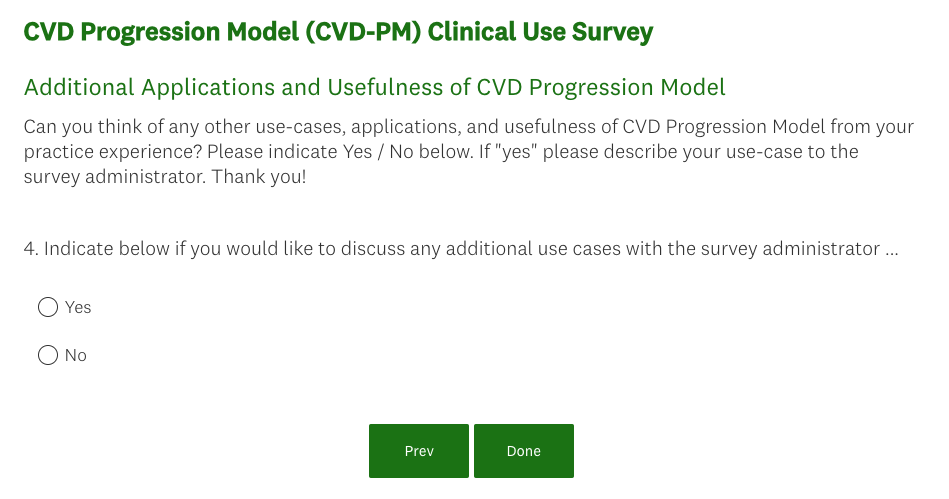


Additional potential application use-cases gathered from Q4 responses:

1. This is really a cohort-level epidemiological analysis and benchmarking tool. The system can be populated with CVD episode data of various clinic cohorts and sub-cohorts (typically 12 to 15 at any point of time) and their patterns (frequency, transition rates, probabilities, etc.) can be compared. This can reveal the trend differences with respect to various cohort control features such as demographics, education, nutrition profile, treatment alternatives etc.
2. Such inter-cohort benchmarking can delineate best practices with respect to cost-effectiveness, manpower economy, fund allocations (e.g., Affordable Care Act incentives) when the candidate cohorts are selected from disparate clinics.
3. The utility can be further extended to understand epidemiological trends of different geographical and population segments, leading to valuable inputs for the healthcare policymakers and administrators enabling them to target specific regions based on pattern differences.
4. This tool can also help comparison of CVD characteristics at a national level, e.g., some countries might have more stroke compared to others having more CHF. This can lead to necessary preventive measures and national health policies.
5. Can be extremely helpful in identifying temporal changes in CVD trends in patients because of shifts in major treatment paradigm (e.g., pre-statin vs. statin period pattern changes)
6. This tool can be potentially integrated with clinic EHR systems to continually monitor temporal pattern shifts over time and send out automated notifications and reports based on preset quality metrics thresholds.

Conclusions:

- Across all questions (Q1, Q2, Q3) there is evidence of strong agreement (Table 2, 81% agree) in regard to the applications and usefulness of CVD-PM within the expert panel
- On the example use-case of CVD-PM as a predictive resource planning tool (Q3), the support is relatively moderate (57% agree)
- In addition to the three use-cases pre-defined in the survey, the open-ended question (Q4) revealed six additional important applications and usefulness of the CVD-PM tool
- Overall, the survey results and comments conclusively led to the characterization of CVD-PM as a “cohort analytics tool” that has many impactful clinical applications and usefulness

**Table S2.** Survey results summary.

|  | **Multiple Choice Questions** | **Agree** | **Somewhat Agree** | **Disagree** | **Total Responses** |
| --- | --- | --- | --- | --- | --- |
| Q1 | This ability will allow clinicians to better understand the effectiveness of the intervention strategies at the clinic cohort level and adjust the quality metrics accordingly. | 6 | 1 | 0 | 7 |
| Percentage Agreement | | 86% | 14% | 0% | 100% |
| Q2 | CVD-PM can provide actionable insights into CVD transition rates for clinic cohorts allowing identification of high-risk patient groups. | 7 | 0 | 0 | 7 |
| Percentage Agreement | | 100% | 0% | 0% | 100% |
| Q3 | CVD-PM can act as an actionable decision support and predictive resource planning tool for the clinic management | 4 | 3 | 0 | 7 |
| Percentage Agreement | | 57% | 43% | 0% | 100% |
| Q1 Q2 Q3 | **Overall agreement across all questions (Q1, Q2, Q3)** | **81%** | **19%** | **0%** | **21** |
|  | | | | | |
|  | **Open-ended Question** | **Yes** | **No** |  | **Total Responses** |
| Q4 | Indicate below if you would like to discuss any additional use cases with the survey administrator | 6 | 1 |  | 7 |
| Percentage Agreement | | 86% | 14% |  | 100% |
