# Supplementary material for: Development of a Cohort Analytics Tool for Monitoring Progression Patterns in Cardiovascular Diseases: Advanced Stochastic Modeling Approach
Source: JMIR Med Inform. 2024 Sep 24;12:e59392. doi: 10.2196/59392 (PMC11462104; doi:10.2196/59392)
Supplement: Multimedia Appendix 7 [file medinform_v12i1e59392_app7.docx]

Artifact for CVD clinicians: The user interface of this 10-state model artifact has an input field (cell with text “Enter current state # here,” Figure 4), where a clinician can enter the current CVD state of a patient under investigation. The artifact automatically calculates and populates all cells with CTMC-based transition probabilities and generates the trend visualization graph for interpretations and appropriate actions, as explained in the CVD Progression Pattern section. This Excel artifact is self-contained and can be downloaded from the code repository at <https://data.mendeley.com/preview/jjx685vj6k>.[1]

Artifacts for future researchers: All underlying source codes and datasets comprising this decision support framework can also be downloaded from the code repository at <https://data.mendeley.com/preview/jjx685vj6k>.[1] A “readme” text file with detailed instructions has been provided that will help future researchers accomplish the following using this framework:

- Reproduce the results published in the Results section of this paper
- Perform additional data analysis on CVD progression based on interest and research goals
- Ingest other CVD datasets from different patient populations to generate new CVD progression models, perform comparative studies, and validate the generalizability
- Ingest patient episode data of other chronic diseases to discover and study their disease progression patterns

Input and output data files, Jupyter Notebook scripts (Python), R Scripts, and the Excel interactive data visualization artifact are available for download at the Mendeley Data site.[1] A README.md file contains details about the files. All data and code are placed in a compressed file (.zip) with a sub-folder structure. When the compressed file is unpacked, it will have the following files and folders, as shown in Figure 1.


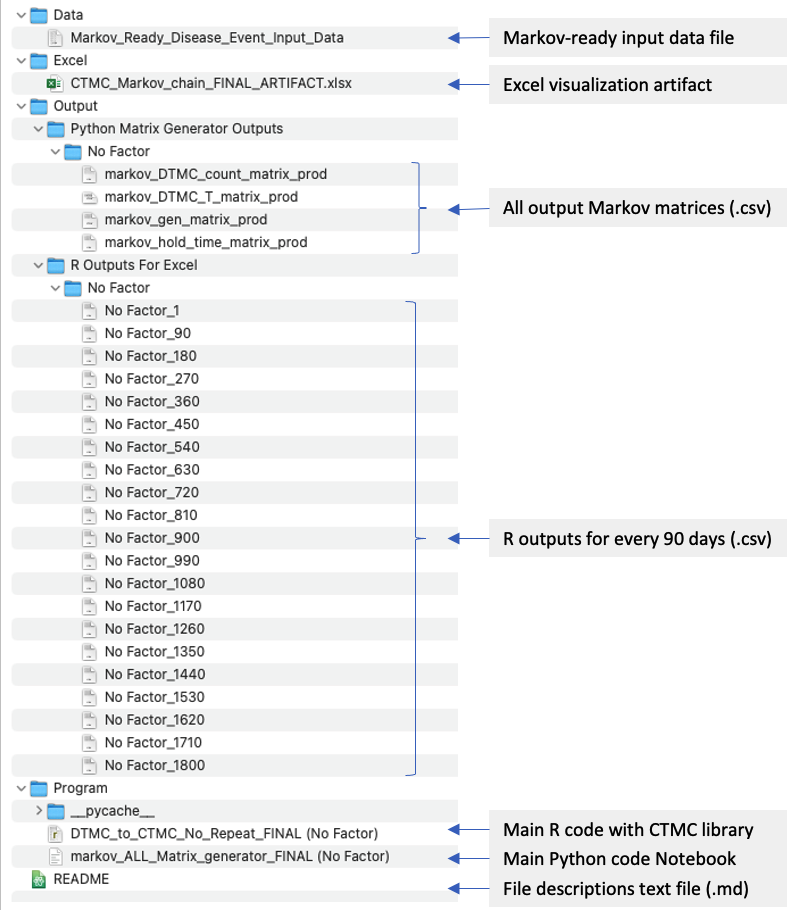


**Figure S1.** System components information for reproducibility.

The programs are coded with the above folder structure in the “path” and are tested to run successfully and produce the results presented in the article without any changes to the code. One can reproduce results by following the steps in Figure 2 in the Methods section.

The code and artifacts provided for the CVD CTMC system can be leveraged with minor modifications to generate CTMC transition matrices for other chronic diseases using the following steps.

Input file mapping: The input data file “Markov_Ready_Disease_Event_Input_Data.csv” has the following data structure (Table 1), and the new chronic disease input data file needs to conform to this format.

**Table S1**. Input data file structure with sample data**^^[[1]](#footnote-1)^^**.

| **nsrrid** | **eventname** | **eventdt** |
| --- | --- | --- |
| 200091 | mi | 568 |
| 200104 | stroke | 1668 |

The chronic disease longitudinal episodic data needs to be pre-processed first to organize in the format as per Table 1, including the exact column names. If the chronic disease has an “absorbing state” such as “death,” it must be mapped to the third column in the code of “markov_ALL_Matrix_generator_FINAL (No Factor).ipynb.” Minor code adjustments might be necessary for mapping the event states of the new chronic disease to those of the CVD code base. The corresponding column names in the code need to be changed accordingly.

After the pre-processing steps are completed, follow the steps in Figure 2 in the Methods section. The programs should run successfully to generate CTMC transition matrices and visualization artifacts.

Reference:

1. Brahma A, Chatterjee S, Tao Y, Seal KC, Fitzpatrick BG. Markov CTMC CVD Progression Framework Compressed Data and Code for JMIR Publication. Mendeley Data2024.

1. nsrrid: an arbitrary identifier that is similar to a unique patient id; eventname: disease episode name; eventdt: number of days from baseline when the episode occurred [↑](#footnote-ref-1)
